# Survival Analysis of Metastatic Early-Onset Colorectal Cancer Compared to Metastatic Average-Onset Colorectal Cancer: A SEER Database Analysis

**DOI:** 10.3390/cancers16112004

**Published:** 2024-05-25

**Authors:** Antoine Jeri-Yabar, Liliana Vittini-Hernandez, Sebastian Prado-Nuñez, Sirish Dharmapuri

**Affiliations:** 1 Department of Medicine, Icahn School of Medicine at Mount Sinai Beth Israel, New York, NY 10029, USA; 2 Department of Medicine, Universidad Peruana Cayetano Heredia, Lima 15102, Peru; 3 Department of Hematology/Oncology, Icahn School of Medicine at Mount Sinai West, New York, NY 10029, USA; sirish.dharmapuri@gmail.com

**Keywords:** early-onset colorectal cancer, overall survival, cancer specific survival, metastatic colorectal cancer

## Abstract

**Simple Summary:**

Over the last decade, early-onset colorectal cancer incidence has continued to increase. The disparities in outcomes and overall survival (OS) between early-onset colorectal cancer and average-onset colorectal cancer remain controversial. The aim of our study was to compare survival rates and identify potential influential factors affecting outcomes between these two groups. Our results show better overall survival and cancer specific survival in patients with early-onset colorectal cancer which could be contributed to better general health, fewer comorbidities and higher likelihood of receiving aggressive cancer treatments.

**Abstract:**

Background: Early-onset colorectal cancer (EO-CRC) is defined as colorectal cancer diagnosed before the age of 50 years, and its incidence has been increasing over the last decade, now accounting for 10% of all new CRC diagnoses. Average-onset colorectal cancer (AO-CRC) has shown a steady decline in its incidence and related mortality over the past 20 years. The disparities in outcomes and overall survival (OS) between EO-CRC and AO-CRC are controversial. Our study compared OS and cause-specific survival (CSS) between metastatic EO-CRC (mEO-CRC) and metastatic AO-CRC (mAO-CRC) and identified the associated factors. Methods: Data on patient characteristics, tumor characteristics, incidence, and mortality were obtained from the SEER database from 2010 to 2020. We identified 23,278 individuals aged > 18 years with a confirmed diagnosis of all histological subtypes of metastatic CRC (M1 on TNM stage) using ICD-O-3 site codes. mEO-CRC and mAO-CRC were compared. OS distributions and CCS were analyzed using the Kaplan–Meier method and log-rank test to assess differences. A Cox regression model was used to assess the associations between variables. Results: mEO-CRC constituted 17.79% of the cases, whereas 82.21% had mAO-CRC. Most patients with mEO-CRC were 45–49 years old (47.66%), male (52.16%) and White (72.57%) and had adenocarcinoma histology (87.30%). Left colon tumors were most prevalent in both groups (40.26%) but were more prevalent in mEO-CRC patients than in mAO-CRC patients (49.63% vs. 38.23%, *p* < 0.001). Patients with mEO-CRC had higher OS (*p* < 0.001) and CSS (*p* < 0.001) than those with mAO-CRC. Patients with mEO-CRC also had significantly better median overall survival (30 months vs. 18 months, *p* < 0.001). The factors associated with worse OS included mAO-CRC (*p* < 0.001), mucinous adenocarcinoma (*p* < 0.001), male sex (*p* = 0.003), and a lack of surgical intervention (*p* < 0.001). Conclusions: Most patients with mEO-CRC fall within the range of 45 to 49 years of age. Patients with mEO-CRC were more likely to receive cancer-directed therapy (including chemotherapy and radiotherapy) and had better OS and CSS than those with mAO-CRC. This is likely attributable to the better performance status, fewer comorbidities, and better tolerance to cancer-directed therapy in mEO-CRC patients. The factors associated with worse OS and CSS were age > 50 years, mucinous adenocarcinoma, male sex, and no surgical treatment.

## 1. Introduction

Colorectal cancer (CRC) is the third most prevalent cancer and second most common cause of cancer-related deaths in the United States [1]. Early-onset colorectal cancer (EO-CRC) is defined as CRC diagnosed before the age of 50 years and is now an emerging health concern [2]. In the last decade, the incidence of early-onset colorectal cancer (EO-CRC) has increased, accounting for 10% of all new CRC diagnoses [3]. This prompted the US Preventive Task Force (USPTF) to decrease the age of CRC screening from 50 to 45 years in 2021 [4]. 

The etiology of EO-CRC is not well known; however, modifiable risk factors, such as excess body weight, diabetes mellitus, alcohol and tobacco use, processed and red meat intake, sugar-sweetened beverage intake, and our microbiota, may play an important role in its pathogenesis [5]. Non-modifiable risk factors associated with EO-CRC include hereditary risk factors such as Lynch syndrome, adenomatous polyposis syndrome, cystic fibrosis, and family history of CRC [6]. Furthermore, the presence of metabolic syndrome increases the risk of EO-CRC by up to 31% in the presence of three comorbid metabolic conditions, including obesity, diabetes mellitus, and dyslipidemia [4]. Regarding survival rates in EO-CRC, patients in the early stages of disease have a favorable prognosis with a five-year survival rate of 90% declining to 71% for stage III and 14% for stage IV [7].

In contrast, average-onset colorectal cancer (AO-CRC) is defined as CRC that presents in patients aged ≥ 50 years [8]. The etiology of AO-CRC has been well studied, with environmental and genetic factors determining the risk of developing CRC—which develops through unique mechanisms such as rapid epithelial cells forming a benign adenoma which can then advance to cancer and metastasize via different pathways including microsatellite instability (MSI) [9]. AO-CRC has shown a steady decline in incidence and related mortality over the past 20 years in the US, which can be attributed to prompt screening modalities and the treatment of premalignant lesions [10]. These divergent epidemiological trends have resulted in a reduction in the median age at diagnosis, from 72 to 66 years [11]. In a study, the five-year overall survival rates for patients with screen-detected CRC stages I, II, and III were 92.4%, 87.9%, and 80.7%, respectively [12]. Regarding stage IV CRC, the three- and five-year overall survival rates were found to be 20.7% and 10.5%, respectively [13]. 

Controversy exists between clinical outcomes in EO-CRC versus AO-CRC, in terms of survival and disease progression risk [14,15]. EO-CRC is considered an independent predictor for worse prognosis, suggesting a potentially more aggressive tumoral phenotype [16,17]. However, other studies have shown that there is a survival benefit for individuals with EO-CRC compared with AO-CRC, potentially due to younger patients being able to receive more aggressive treatment due to better tolerance, undergoing metastases resection more often and better ECOG scale performance as compared to patients >50 years of age who are more prone to have other comorbidities as well [18]. The aim of this study is to evaluate and compare the survival differences between metastatic early-onset colorectal cancer (mEO-CRC) and metastatic average-onset colorectal cancer (mAO-CRC) and identify associated factors. 

## 2. Methods

### 2.1. Population 

**Figure 1:**
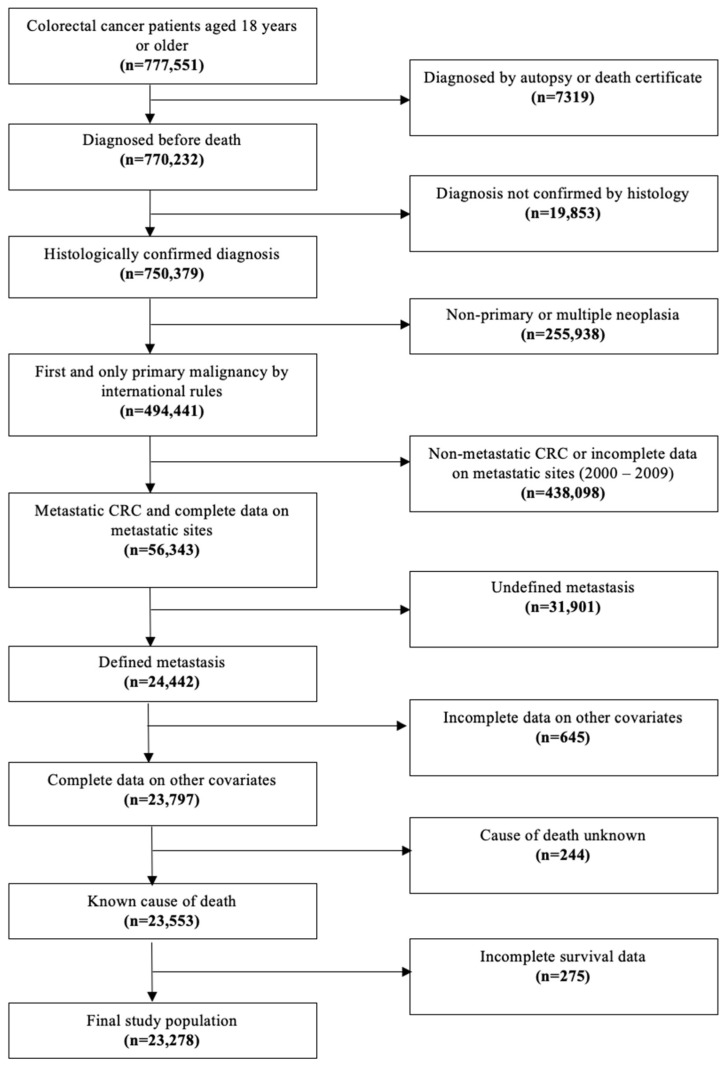
Flow diagram for selection of the study cohort.

We evaluated patients ≥18 years old with metastatic colorectal cancer from the Surveillance, Epidemiology, and End Results (SEER) database with a study period from 2010 to 2020 [19]. The SEER database includes data on cancer incidence, survival, extent of disease, and treatment for 30% of the United States population; SEER has collected information regarding sites of metastasis since 2010; hence, our study period ranges from 2010 to 2020. The study population included adult patients diagnosed with metastatic colorectal cancer (n = 23,278), and we divided them into two groups defined by age: <50 years of age or mEO-CRC (n = 4141) and >50 years of age or mAO-CRC (n = 19,137). Individuals diagnosed at age 50 were excluded from the study due to the possibility of screening for detection bias. The final study population was 23,278. Other inclusion factors were histologically confirmed diagnoses including specific ICD-O-3 site codes (C180, C182-9, C209, and histology codes: 8000, 8010, 8012, 8013, 8020-2, 8031-33, 8041, 8045, 8070-2, 8083, 8123-4, 8140, 8144-45, 8201, 8210-11, 8213, 8220-1, 8240, 8243-46, 8249, 8253, 8255, 8260-3, 8261-3, 8265, 8310, 8323, 8480-1, 8490, 8507, 8510, 8550, 8560, 8570, 8574, 8936, and 8980), first and only malignancy, confirmed metastatic CRC, complete data on metastatic sites, and known cause of death. Patients with incomplete information regarding undefined metastasis, unknown cause of death, or incomplete survival data were excluded from the study (Figure 1). 

### 2.2. Study Design and Primary Outcome 

A retrospective cohort study of survival analysis was performed. Overall survival was the primary outcome and was defined as the time from cancer diagnosis to death. Cancer-specific survival was defined as the time from diagnosis to death from metastatic colorectal cancer only; patients who died of other causes were censored from the cancer-specific survival analysis.

### 2.3. Statistical Analysis

The databases used were downloaded using the SEERStat v8.4.2 interface and exported to the statistical package STATA v16.0 [20] to conduct all analyses. Categorical variables were described as frequencies and percentages, whereas continuous variables were described as mean and standard deviation or median with interquartile range (IQR). Categorical variables were compared using the chi-squared test, whereas numeric variables were compared using the Mann–Whitney U test after assessing normality.

The Kaplan–Meier method was used to evaluate survival or event absence, and the log-rank test was used to assess differences between groups. Additionally, univariate and multivariate Cox regression models were used to assess the association between the exposure variables and all-cause mortality. Hazard ratios (HR) were reported as crude and adjusted with 95% confidence intervals (95% CI). The multivariate model included adjustment variables with *p* < 0.05 using the backward selection method, such as sex, N score, histological grade, surgical treatment, radiation, and chemotherapy. Multicollinearity was evaluated using variance inflation factors (VIF) with a cutoff point set at less than 5. Finally, a *p*-value < 0.05 was considered statistically significant.

## 3. Results

Among 23,278 individuals with metastatic CRC, 17.79% were diagnosed with mEO-CRC, and 82.21% were diagnosed with mAO-CRC. Most individuals in the study were male (52.71%), and White race was predominant (75.67%). Among mEO-CRC cases, the highest prevalence was observed in patients aged 45–49 (47.66%), with the next highest prevalence seen in those aged 40–44 (26.08%). The most common histologic subtype was adenocarcinoma (87.37%), followed by mucinous adenocarcinoma (7.62%), in both mEO-CRC and mAO-CRC. In both groups, left colon tumors were the most common primary site; however, they comprised a higher percentage of cases in mEO-CRC cases (49.63%) than in mAO-CRC cases (38.23%). The right colon, as the primary tumor site, constituted 12.29% of all mEO-CRC cases and 18.10% of mAO-CRC cases. Most patients in both groups underwent surgery (88.38%) and chemotherapy (70.32%). However, chemotherapy was more common in the mEO-CRC group (87.23%) than in the mAO-CRC group (66.66%) (*p* < 0.001). Additional descriptive information for the individuals by age group is presented in Table 1. 

Regarding patterns of distant metastasis, the majority of the study population (93.32%) exhibited solitary site metastasis. The most common site of metastasis was the liver for both mEO-CRC (70.06%) and mAO-CRC (69.97%) patients. However, mAO-CRC patients appeared to have a higher incidence of lung-only metastasis than mEO-CRC patients at the time of diagnosis (19.33% vs. 16.76%; *p* < 0.001). In addition, mAO-CRC patients had a higher incidence of brain metastasis (1.03%, *p* = 0.004) and bone-only metastases (3.63%; *p* = 0.036) than mEO-CRC patients (0.56% and 2.97%, respectively). Detailed metastatic site data are presented in Table 2. 

**Figure 2:**
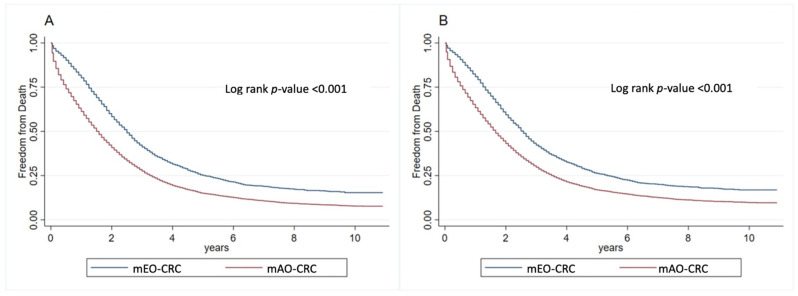
Overall survival (**A**) and specific survival (**B**) of mEO-CRC patients compared with mAO-CRC patients.

**Figure 3:**
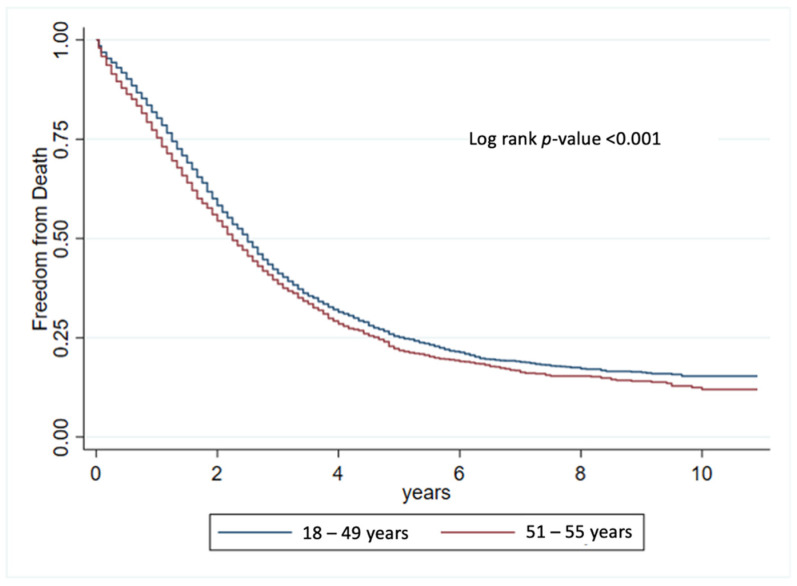
Overall survival in patients with EO-CRC vs. sub-stratified AO-CRC (51–55 years old).

**Figure 4:**
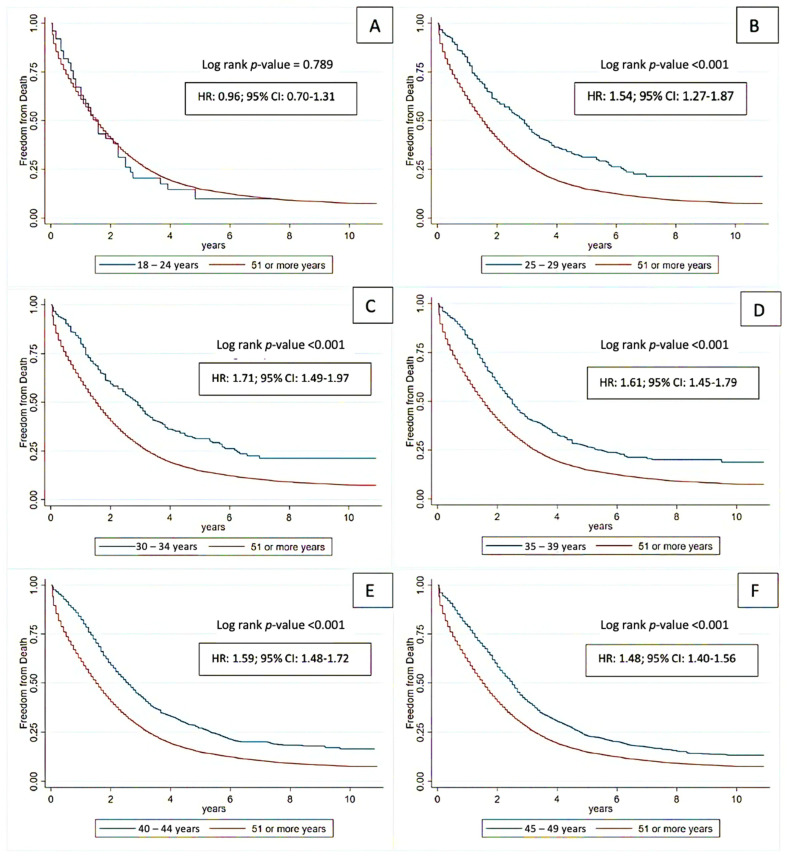
Overall survival in patients with CRC stratified by age groups. (**A**): 18–24 years vs. 51 or more; (**B**): 25–29 vs. 51 or more; (**C**): 30–34 years vs. 51 or more, (**D**): 35–39 years vs. 51 or more; (**E**): 40–44 vs. 51 or more; (**F**): 45–49 vs. 51 or more.

The overall survival and cancer-specific survival rates are shown in Figure 2. Compared with individuals diagnosed with CRC at ages above 50 years, individuals with mEO-CRC had a higher overall survival rate (log-rank *p* < 0.001) as well as a higher cancer-specific survival rate (log-rank *p* < 0.001). Moreover, an additional survival analysis was conducted to compare mEO-CRC (18–49 years) with a subgroup of mAO-CRC (ages 51–55). This analysis aimed to mitigate potential confounding factors in overall survival, given the higher likelihood of other comorbidities in patients aged > 55 years, which could potentially influence overall survival outcomes (Figure 3). These results were similar to those shown in Figure 2 with a *p*-value < 0.001. Furthermore, we subdivided the mEO-CRC patients into different age groups and compared the survival outcomes between the various subgroups of mEO-CRC and mAO-CRC, where a trend of better survival was evidenced in all subgroups of mEO-CRC when compared to mAO-CRC, except for the group of ages 18–24 versus 51 years of age or older; however, the 18–24 group has a very small sample size (n = 50) and therefore limited statistical power (Figure 4). 

We further analyzed survival differences between mEO-CRC and mAO-CRC patients, adjusting for other predictors associated with mortality (Table 3 and Table 4). Compared with adenocarcinoma, mucinous adenocarcinoma had an increased HR of 1.17 (*p* < 0.001) of mortality. Individuals with T stage T2–T3 had an HR of 0.53 and 0.64, respectively, with *p* < 0.001 showing a reduction in mortality, and N2 had an adjusted HR of 1.59 (*p* < 0.001). Treatment with chemotherapy and surgery showed a reduction in mortality (HR 0.48, *p* < 0.001), as well as chemotherapy, surgery, and radiotherapy (HR 0.81, *p* < 0.001). Compared with mAO-CRC patients, mEO-CRC patients also had significantly better median overall survival (30 months vs. 18 months, *p* < 0.001).

## 4. Discussion

The escalating incidence of early-onset colorectal cancer (EO-CRC) poses a burgeoning challenge, and although its etiology is not fully understood, it is suspected to be multifactorial [4,6]. The multifactorial nature of the rise in EO-CRC cases has some identifiable risk factors [17]. For example, external exposure from an early age from conception to adulthood to a sedentary lifestyle, diet, socioeconomic background, antibiotic exposure, and intrinsic factors such as genetics, oxidative stress, and gut microbiota play an important role in pathogenesis [21]. These forms of exposure can cause genetic and epigenetic alterations in epithelial cells and affect gut microbiota and host immunity [22]. A recent study on the EO-CRC intestinal microbiome and host–microbe interaction showed a significant difference in species enrichment between EO-CRC and AO-CRC, along with stronger microbe–host interactions in EO-CRC vs. AO-CRC at the tumor site, suggesting a direct role of microbes in the genesis of the tumor via cancer-related pathways [23,24]. Despite these associations, many underlying mechanisms remain unclear [4].

The existing literature presents discordance in findings regarding overall survival and outcomes in EO-CRC as compared to AO-CRC [13,14,16,17]. Notably, a Canadian study revealed elevated survival rates in EO-CRC patients, specifically within the age range of 40–49 years, whereas diminished survival rates were observed outside this specified age bracket [3]. In contrast, a comprehensive review conducted by Chang et al. indicated that despite histological features suggestive of a poorer prognosis, EO-CRC patients exhibited superior overall and disease-free survival rates compared to AO-CRC patients [25]. However, a nuanced analysis stratified by age group revealed a generally poor prognosis for patients aged < 35 years old [7]. Moreover, another study indicated that the recurrence/progression-free survival and cancer-specific survival of EO-CRC in stage I surgical candidates are inferior to those of AO-CRC patients, with EO-CRC also being an independent predictor for worse prognosis [16]. 

Our study highlights improved overall survival in mEO-CRC patients compared to mAO-CRC patients, a finding that remains consistent across most sub-stratified age groups. Multiple factors could have played a role in these results, including more aggressive therapies and fewer comorbidities in the mEO-CRC group [26]. In addition, increased utilization of multimodality therapy, including surgical metastasectomy and radiation, immunotherapy for MSI-H tumors, and third-line chemotherapy, have been associated with improved survival in CRC [27,28]. A study comparing data from three prospective randomized European trials regarding survival after a second liver resection in metastatic colorectal cancer patients showed that a younger patient cohort and improved resection techniques may have had a positive impact on survival in the LICC trial, which included a younger population than in other trials [29]. Furthermore, the mEO-CRC cohort generally had fewer comorbidities and a better performance status, which may correlate with the administration of more aggressive chemotherapy and radiotherapy by oncologists in comparison to mAO-CRC [26], including decreased breaks between treatments. In line with the existing literature, our study demonstrated a higher likelihood of receiving chemotherapy in mEO-CRC patients (87.23% vs. 66.6%), potentially attributed to their better tolerance to the chemotherapy regimen, including multiagent therapy, than older patients [30]. It is important to note, however, that EO-CRC appears to be genomically indistinguishable from AO-CRCs; therefore, treatment options do not differ, and further aggressive treatment regimens based solely on age are not warranted [31]. It should also be noted that our study did not reveal improved survival for the subgroup aged 18–24 compared to those aged 51 years or older. This outcome was likely due to the limited statistical power stemming from the small sample size of this subgroup (n = 50).

To address potential confounding factors in the overall survival comparison, such as comorbidities and increased mortality from other causes, a distinct subgroup of mAO-CRC was delineated, comprising individuals aged 51 to 55 years. Notably, our initial Kaplan–Meier curve encompassed all patients over 50 years of age. Analysis of overall survival and specific survival in mEO-CRC versus mAO-CRC revealed a pronounced increase in survival rates among mEO-CRC patients, even when compared to a subset of the mAO-CRC population. These findings align with the outcomes reported in a JAMA study that investigated overall survival in EO-CRC and AO-CRC patients aged 51–55 years of age [14]. Importantly, our study diverges from prior research as it comprehensively examines survival disparities between mEO-CRC and mAO-CRC across the entire mAO-CRC population and a presumably “healthier” subgroup. The results consistently indicate superior overall survival in patients with mEO-CRC, offering valuable insights into the distinctive dynamics of colorectal cancer survival across age groups.

In the United States, the colorectal cancer (CRC) screening age has been reduced from 50 to 45 years, with the aim of eliminating premalignant lesions and detecting the disease at early stages in asymptomatic individuals [4]. Some countries have even lowered their screening age to 40 years [32]. Despite this measure, approximately one-third of patients will not undergo screening in the general screening process, as they present with the disease before the age of 45 [7]. The study, based on the SEER database, lacks epidemiological information on potential risk factors, but it is noted that physical inactivity, high-calorie diets, type 2 diabetes mellitus, and smoking are described as contributing factors [6] and further research must be conducted for early identification of patients younger than 50 years of age who would benefit from early CRC screening due to them being at high risk; a risk scoring system to predict the individual incidence of early-onset colorectal cancer is being studied and would be of great benefit in the primary care setting [33].

This study has several limitations, including the absence of complete epidemiological data to fully characterize the population and the non-prospective nature of the study. Additionally, there is no information on the genomic characteristics of tumors, which are of great interest in mEO-CRC and pave the way for targeted management. Notably, the study did not specify the treatment administered or whether it was intended for curative or palliative purposes. Consequently, it is challenging to ascertain whether the survival of mEO-CRC patients is improved by more intensive treatment or the natural course of the disease. Additionally, it must be addressed that this study included data from 2020, when the COVID-19 pandemic was ongoing, which could have prevented prompt treatment in some patients and therefore this could have impacted survival. Additionally, our study encompasses all anatomical locations within the colon and rectum. We intentionally adopted a generalized approach to broadly compare survival outcomes between mEO-CRC and mAO-CRC patients. This approach was chosen to provide a foundational understanding of survival differences across a wide demographic and disease spectrum, which could then inform more detailed, targeted studies. Finally, as an observational study, it was subject to the inherent limitations associated with this study type. Nevertheless, this study effectively characterizes mEO-CRC, highlighting key distinctions from mAO-CRC.

## 5. Conclusions

EO-CRC is a progressively increasing entity that is often detected in advanced stages owing to the lack of specific screening in asymptomatic young population groups. The overall survival of mEO-CRC patients is superior to that of mAO-CRC patients, not only across the entire group but also within the younger population of mAO-CRC. These findings can be explained by the mEO-CRC cohort having a better performance status, fewer comorbidities, better tolerance to multimodality chemotherapy, and higher use of multimodality therapy, including radiotherapy and surgery. Although OS appears to be superior in mEO-CRC, it remains crucial to identify potential candidates that could benefit from early CRC screening to diagnose the disease at its initial stages. The development of risk-scoring systems is essential to identify this subset of high-risk individuals, ultimately enhancing early detection and improving clinical outcomes.

## Figures and Tables

**Table 1:** General characteristics of the study population.

Characteristics	Entire Sample (n = 23,278)		Early-Onset (n = 4141)		Average-Onset (n = 19,137)		*p*-Value
	n	%	n	%	n	%	
Age (years)							
18–24	50	0.21	-	-	-	-	
25–29	151	0.65					
30–34	329	1.41					
35–39	558	2.40					
40–44	1080	4.64					
45–49	1973	8.48					
51 or older	19,137	82.21	-	-	-	-	
Sex							0.438 ^†^
Female	11,009	47.29	1981	47.84	9028	47.18	
Male	12,269	52.71	2160	52.16	10,109	52.82	
Race							<0.001 ^†^
White	17,615	75.67	3001	72.47	14,614	76.37	
Black	3314	14.24	632	15.26	2682	14.01	
Asian/Pacific Islander	2138	9.18	451	10.89	1687	8.82	
American Indian/Alaska Native	211	0.91	57	1.38	154	0.80	
Histologic subtype							0.596 ^†^
Adenocarcinoma	20,399	87.37	3615	87.30	16,724	87.39	
Mucinous adenocarcinoma	1773	7.62	308	7.43	1465	7.66	
Large-cell neuroendocrine carcinoma	511	2.20	89	2.15	422	2.21	
Others	655	2.81	129	3.12	526	2.75	
Tumor size (mm)							0.394 ^‡^
Median (IQR)	50 (40–70)		50 (40–70)		50 (40–70)		
T score							0.677 ^†^
T1	1038	4.46	183	4.42	855	4.47	
T2	666	2.86	121	2.92	545	2.85	
T3	11,506	49.43	2080	50.23	9426	49.25	
T4	10,068	43.25	1757	42.43	8311	43.43	
N score							<0.001 ^†^
N0	4342	18.65	649	15.67	3693	19.30	
N1	8907	38.26	1552	37.48	7355	38.43	
N2	10,029	43.08	1940	46.85	8089	42.27	
Histologic grade							0.065 ^†^
Well differentiated	1149	4.94	198	4.78	951	4.97	
Moderately differentiated	15,037	64.60	2749	66.38	12,288	64.21	
Poorly differentiated	5932	25.48	995	24.03	4937	25.80	
Undifferentiated	1160	4.98	199	4.81	961	5.02	
Primary tumor site							<0.001 ^†^
Right colon	3973	17.07	509	12.29	3464	18.10	
Left colon	9372	40.26	2055	49.63	7317	38.23	
Transverse colon	1676	7.20	249	6.01	1427	7.46	
Cecum	5076	21.80	552	13.33	4524	23.64	
Rectum	3181	13.67	776	18.74	2405	12.57	
Surgical treatment							0.140 ^†^
No/Unknown	2706	11.62	509	12.29	2197	11.48	
Yes	20,572	88.38	3632	87.71	16,940	88.52	
Radiotherapy							<0.001 ^†^
No/Unknown	20,841	89.53	3523	85.08	17,318	90.49	
Yes	2437	10.47	618	14.92	1819	9.51	
Chemotherapy							<0.001 ^†^
No/Unknown	6909	29.68	529	12.77	6380	33.34	
Yes	16,369	70.32	3612	87.23	12,757	66.66	
Vital Status							<0.001 ^†^
Alive	5565	23.91	1348	32.55	4217	22.04	
Died	17,713	76.09	2793	67.45	14,920	77.96	
Follow-up (years)							<0.001 ^‡^
Median (IQR)	1.42 (0.50–2.83)		2 (1.00–3.67)		1.25 (0.42–2.67)		

IQR: interquartile range. ^†^ Chi-squared test; ^‡^ U Mann–Whitney test.

**Table 2:** Metastatic site combinations by early- and average-onset in adults with metastatic colorectal cancer at diagnosis.

Metastasis Site	Early-Onset		Average-Onset		*p*-Value ^†^
	n	%	n	%	
One site					
Only liver	2901	70.06	13,391	69.97	0.918
Only lung	694	16.76	3700	19.33	<0.001
Only bone	123	2.97	695	3.63	0.036
Only brain	23	0.56	197	1.03	0.004
Two sites					
Liver and lung	455	10.99	2376	12.42	0.011
Liver and bone	85	2.05	470	2.46	0.123
Liver and brain	10	0.24	90	0.47	0.041
Lung and bone	49	1.18	259	1.35	0.385
Lung and brain	10	0.24	75	0.39	0.146
Bone and brain	7	0.17	33	0.17	0.962
Three sites					
Liver and lung and bone	35	0.85	197	1.03	0.279
Liver and lung and brain	7	0.17	49	0.26	0.300
Liver and bone and brain	5	0.12	24	0.13	0.938
Lung and bone and brain	5	0.12	17	0.09	0.545
Four sites					
Liver and lung and bone and brain	3	0.07	14	0.07	0.988

^†^ Chi-squared test.

**Table 3:** Multivariate analysis of the relationship between early- and average-onset metastatic colorectal cancer and all-cause mortality.

Exposure	Crude Model ^a^			Adjusted Model ^a,b^		
	HR	95% CI	*p*-Value	HR	95% CI	*p*-Value
Age						
Early onset	Ref.	---	---	Ref.	---	---
Average onset	1.53	1.47–1.59	<0.001	1.36	1.31–1.42	<0.001
Sex						
Female	Ref.	---	---	Ref.	---	---
Male	1.01	0.97–1.03	0.818	1.05	1.02–1.08	0.003
Race						
White	Ref.	---	---	Not evaluated ^†^		
Black	1.08	1.04–1.13	<0.001			
Asian/Pacific Islander	0.89	0.84–0.94	<0.001			
American Indian/Alaska Native	0.95	0.81–1.11	0.522			
Histologic subtype				Not evaluated ^†^		
Adenocarcinoma	Ref.	---	---			
Mucinous adenocarcinoma	1.17	1.11–1.24	<0.001			
Large cell neuroendocrine carcinoma	1.11	1.01–1.23	0.035			
Others	1.67	1.54–1.82	<0.001			
T score				Not evaluated ^‡^		
T1	Ref.	---	---			
T2	0.53	0.47–0.59	<0.001			
T3	0.64	0.60–0.68	<0.001			
T4	0.92	0.86–0.99	0.002			
N score						
N0	Ref.	---	---	Ref.	---	---
N1	1.04	1.00–1.09	0.057	1.18	1.13–1.23	<0.001
N2	1.36	1.30–1.41	<0.001	1.59	1.53–1.67	<0.001
Histologic grade						
Well differentiated	Ref.	---	---	Ref.	---	---
Moderately differentiated	1.10	1.03–1.18	0.008	1.31	1.22–1.41	<0.001
Poorly differentiated	1.75	1.63–1.89	<0.001	1.97	1.83–2.13	<0.001
Undifferentiated	1.90	1.73–2.08	<0.001	2.11	1.92–2.31	<0.001
Surgical treatment						
No/Unknown	Ref.	---	---	Ref.	---	---
Yes	0.64	0.61–0.66	<0.001	0.46	0.44–0.48	<0.001
Radiotherapy						
No/Unknown	Ref.	---	---	Ref.	---	---
Yes	0.71	0.68–0.75	<0.001	0.81	0.77–0.85	<0.001
Chemotherapy						
No/Unknown	Ref.	---	---	Ref.	---	---
Yes	0.36	0.35–0.38	<0.001	0.35	0.34–0.36	<0.001

HR: hazard ratio; 95% CI: 95% confidence intervals. ^a^ Cox regression model; ^b^ Adjusted for age, sex, N score, histologic grade, surgical treatment, radiation, and chemotherapy. ^†^ Variables that did not enter the adjusted regression model because they showed a *p*-value > 0.05 in the crude regression model; ^‡^ Variables that did not enter the adjusted regression model because they presented collinearity with other variables.

**Table 4:** Multivariate analysis of the relationship between metastatic colorectal cancer treatment and all-cause mortality.

Exposure	Crude Model ^a^			Adjusted Model ^a,b^		
	HR	95% CI	*p*-Value	HR	95% CI	*p*-Value
Treatment						
QT	Ref.	---	---	Ref.	---	---
QT + Qx	0.55	0.52–0.58	<0.001	0.48	0.45–0.52	<0.001
Treatment						
QT + Qx	Ref.	---	---	Ref.	---	---
QT + Qx + RT	0.71	0.66–0.75	<0.001	0.81	0.76–0.87	<0.001

HR: hazard ratio; 95% CI: 95% confidence intervals; QT: chemotherapy; Qx: surgery; RT: radiotherapy. ^a^ Cox regression model; ^b^ adjusted for age, sex, histologic subtype. tumor size, T and N score, and histology grade.

## Data Availability

Research data supporting this publication are available.
